# Millennials and Their Parents: Implications of the New Young Adulthood for Midlife Adults

**DOI:** 10.1093/geroni/igx026

**Published:** 2017-11-20

**Authors:** Karen L Fingerman

**Affiliations:** Department of Human Development and Family Sciences, The University of Texas at Austin

**Keywords:** Emerging adulthood, intergenerational relationships, intergenerational support, life course theory, transition to adulthood

## Abstract

The period of young adulthood has transformed dramatically over the past few decades. Today, scholars refer to “emerging adulthood” and “transitions to adulthood” to describe adults in their 20s. Prolonged youth has brought concomitant prolonged parenthood. This article addresses 3 areas of change in parent/child ties, increased (a) contact between generations, (b) support from parents to grown children as well as coresidence and (c) affection between the generations. We apply the *Multidimensional Intergenerational Support Model* (MISM) to explain these changes, considering societal (e.g., economic, technological), cultural, family demographic (e.g., fertility, stepparenting), relationship, and psychological (normative beliefs, affection) factors. Several theoretical perspectives (e.g., life course theory, family systems theory) suggest that these changes may have implications for the midlife parents’ well-being. For example, parents may incur deleterious effects from (a) grown children’s problems or (b) their own normative beliefs that offspring should be independent. Parents may benefit via opportunities for generativity with young adult offspring. Furthermore, current patterns may affect future parental aging. As parents incur declines of late life, they may be able to turn to caregivers with whom they have intimate bonds. Alternately, parents may be less able to obtain such care due to demographic changes involving grown children raising their own children later or who have never fully launched. It is important to consider shifts in the nature of young adulthood to prepare for midlife parents’ future aging.

**Translational Significance**

Clinicians will be able to help normalize situations when midlife parents are upset due to involvement with their young adult children. Policy makers may be able to foresee and plan for future issues involving aging parents and midlife children.

Young adulthood has changed dramatically since the middle of the 20th century. Research over the past two decades has documented this restructuring, relabeling the late teens and 20s under the auspices of “transitions to adulthood” or “emerging adulthood” (Arnett, 2000; Furstenberg, 2010). As such, the life stage from ages 18 to 30 has shifted from being clearly ensconced in adulthood, to an interim period marked by considerable heterogeneity. Historically, young people also took circuitous paths in their careers and love interests (Keniston, 1970; Mintz, 2015), but a recent U.S. Census report shows that young people today are less likely to achieve traditional markers of adulthood such as completion of education, marriage, moving out of the parental home or securing a job with a livable wage as they did in the mid to late twentieth century (Vespa, 2017). Individuals who achieve such markers do so at later ages, and patterns vary by socioeconomic background (Furstenberg, 2010).

Much of the research regarding this stage of life has focused on antecedents of young adult pathways or implications of different transitions for the young adults’ well-being (Schulenberg & Schoon, 2012). Yet, the prolongation of entry into adulthood involves a concomitant prolongation of midlife parenthood; implications of parenting young adult offspring remain poorly understood. This article focuses on midlife parents’ involvement with grown children from the parents’ perspective (and does not address implications for grown children).

Several theoretical perspectives suggest that parents will be affected by changes in the nature of young adulthood. The life course theory concept “linked lives” suggests that events in one party’s life influence their close relationship partners’ lives. Family systems theory posits that changes in one family member’s life circumstances will reverberate throughout the family, even when children are grown (Fingerman & Bermann, 2000). Further, the developmental stake hypothesis suggests that parents’ high investment and involvement with young adult children may generate both a current and a longer term impact on parental well-being (Birditt, Hartnett, Fingerman, Zarit, & Antonucci, 2015). These theories collectively suggest that events in young adults’ lives may reverberate through their parents’ lives.

As such, this article addresses changes that midlife parents experience stemming from shifts in young adulthood. Specifically it describes (a) what has changed in ties between midlife parents and young adults over the past two decades, (b) why these changes have occurred, and (c) the implications of these changes for parents’ well-being currently in midlife, and in the future if they incur physical declines, cognitive deficits, or social losses associated with late life.

## What Has Changed in Parents’ Ties to Young Adults

Parental involvement with young adult children has increased dramatically over the past few decades. Notably, there has been an increase in parents’ contact with, support of, coresidence, and intimacy with young adult children (Arnett & Schwab, 2012a; Fingerman, 2016; Fingerman, Miller, Birditt, & Zarit, 2009; Fry, 2016; Johnson, 2013).

### Parental Contact With Young Adult Children

Parents have more frequent contact with their young adult children than was the case thirty years ago. Research using national US data from the mid to late twentieth century revealed that only half of parents reported contact with a grown child at least once a week (Fingerman, Cheng, Tighe, Birditt, & Zarit, 2012). Because most parents have more than one grown child, by inference many grown children had even less frequent contact with their parents. Recent studies in the twenty-first century, however, found that nearly all parents had contact with a grown child in the past week, and over half of parents had contact with a grown child everyday (Arnett & Schwab, 2012b; Fingerman, et al., 2016).

It would be remiss to imply that all midlife parents have frequent contact with their grown children, however, because a small group shows the opposite trend. From the child’s perspective, national data reveal 20% of young adults lack contact with a father, and 6.5% lack contact with a mother figure in the United States (Hartnett, Fingerman, & Birditt, 2017). Similarly, research examining Lesbian, gay, bisexual, and transgender (LGBT) young adults suggests that some parents reject grown children who declare a minority sexuality or gender identity, but this appears to be a relatively rare occurrence. Instead, a representative survey found that LGBT young adults choose whether to come out to parents; only 56% had told their mother and only 39% had told their father (Pew Research Center, 2013). As such, it seems that LGBT young adults who are likely to be rejected by parents may decide not to tell them about their sexuality. Death accounted for some of the lack of parents (4% of young adults lack a father due to death and 3% lack a mother). Rather, divorce, incarceration, and other factors such as addiction or earlier placement in foster care may account for estrangement from a parent figure (Hartnett et al., 2017). Of course, estrangement may be different from the parents’ perspective. For example, one study of aging mothers found that 11% of aging mothers reported being estranged from one child (Gilligan, Suitor, & Pillemer, 2015), but these mothers rarely reported being estranged from all of their children. Nevertheless, a significant subgroup of parents may be excluded from increased involvement described here for other parents.

### Parental Support of Young Adult Children

Parents also give more support to grown children, on average, than parents gave in the recent past. Across social strata, parents provide approximately 10% of their income to young adult children, a shift from the late twentieth century (Kornich & Furstenberg, 2013). From the 1970s through the 1990s, parents spent the most money on children during the teenage years. But since 2000, parents across economic strata have spent the most money on children under age 6 or young adult children over the age of 18 (Kornich & Furstenberg, 2013). Indeed, some scholars have suggested that over a third of the financial costs of parenting occur after children are age 18 (Mintz, 2015).

The amount of financial support parents provide varies by the parents’ and grown child’s SES, however. Parents from higher socioeconomic strata provide more financial assistance to adult children (Fingerman et al., 2015; Grundy, 2005). This pattern is not limited to the United States; better off parents invest money in young adult offspring who are pursuing education or who have not yet secured steady employment in most industrialized nations (Albertini & Kohli, 2012; Fingerman et al., 2016; Swartz, Kim, Uno, Mortimer, & O’Brien, 2011). Yet, this pattern may perpetuate socioeconomic inequalities in the United States, rendering lower SES parents more likely to have lower SES grown children (Torche, 2015).

In addition to financial support, many parents devote time to grown children (e.g., giving practical or emotional support; Fingerman et al., 2009). Young people face considerable demands gaining a foothold in the adult world (e.g., education, jobs, evolving romantic ties; Furstenberg, 2010). In response, parents may offer adult offspring help by making doctor’s appointments, or giving advice and emotional support at a distance, using phone, video technologies, text messages, or email.

Such nonmaterial support may stem from early life patterns. In early life, parenting has become more time intensive over the past few decades, particularly among upper SES parents (Bianchi & Milkie, 2010). Lower SES parents may work multiple jobs or face constraints (e.g., rigid work hours, multiple shifts) that preclude intensive parenting more typical in upper SES families (Conger, Conger, & Martin, 2010). It is not clear whether such differences in time persist in adulthood.

Rather, the types of nonmaterial support may differ by SES. Research suggests better off parents are more likely to give information and to spend time listening to grown children, and less well-off parents provide more childcare (i.e., for their grandchildren; Fingerman et al., 2015; Henretta, Grundy, & Harris, 2002). Grown children in better off families are more likely to pursue higher education, and student status is strongly associated with parental support (including time as well as money) throughout the world (Fingerman et al., 2016; Henretta, Wolf, van Voorhis, & Soldo, 2012). Yet, less well-off parents are more likely to coreside with a grown child.

Nevertheless, research suggests that across SES strata, midlife parents attempt to support grown children in need. A recent study found that overall, lower SES parents gave as much or more support than upper SES parents, but lower SES young adult children were still likely to receive less support on average (i.e., due to greater needs across multiple family members in lower SES families; Fingerman et al., 2015).

### Parental Coresidence With Young Adult Children

Coresidence could be conceptualized as a form of support from parents to grown children; grown children who reside with parents save money and may receive advice, food, childcare or other forms of everyday support. In industrialized nations, rates of intergenerational coresidence have risen in the past few decades. In the United States in 2015, intergenerational coresidence became the modal residential pattern for adults aged 18 to 34, surpassing residing with romantic partners for the first time (Fry, 2015, 2016). Rates of coresidence have increased in many European countries as well in the past 30 years, though rates vary by country. Coresidence is common in Southern European nations (e.g., 73% of adults aged 18 to 34 lived with parents in Italy in 2007), but relatively rare in Nordic nations (e.g., 21% of young adults lived with parents in Finland in 2007). Coresidence rates in Southern European countries evolved from historical patterns, but also reflect an increase over the past 40 years. For example, in Spain in 1977, fewer than half of young adults remained in the parents’ home, but by the early 21st century over two-thirds of young adults did (Newman, 2011). Coresidence appears to be an extension of the increased involvement between adults and parents (as well as reflecting offspring’s economic needs).

### Parental Affection, Solidarity, and Ambivalence Towards Young Adult Children

In general, affection between young adults and parents seems to be increasing in the twenty-first century as well. It is not possible to objectively document changes in the strength of emotional bonds due to measurement issues and ceiling effects—most people have reported close ties to parents or grown children across the decades. Still, it seems intergenerational intimacy is on the rise. In the 20th century in Western societies, marriage was the primary tie. Yet, over 15 years ago, Bengtson (2001) speculated that the prominence of multigenerational ties would rise in the 21st century due to changes in family structure (e.g., dissolution of romantic bonds) and longevity (e.g., generations sharing more years together). Bengtson’s predictions seem to be coming to fruition.

Increases in midlife parents’ affection for young adult children would be consistent with a rise in intergenerational solidarity. *Intergenerational solidarity theory* was developed in the 20th century to explain strengths in intergenerational bonds (Bengtson, 2001; Lowenstein, 2007). Solidarity theory is mechanistic in nature, suggesting that positive features of relationships (e.g., contact, support, shared values, affection) co-occur like intertwining gears. In this regard, we might conceptualize the overall increase in parental involvement as increased intergenerational solidarity.

It is less clear whether conflictual or negative aspects of the relationship have changed in the past few decades. It was only towards the end of the 20th century that researchers began to measure ambivalence (mixed feelings) or conflict in this tie (Fingerman, 2001; Luescher & Pillemer, 1998; Pillemer et al., 2007; Suitor, Gilligan, & Pillemer, 2014). As such, it is difficult to track changes in ambivalence across the decades. Nevertheless, one study found that midlife adults experienced greater ambivalence or negative feelings for their young adult children than for their aging parents (Birditt et al., 2015), suggesting the parent/child tie may have shifted towards greater ambivalence in that younger generation.

Indeed, scholars have argued that ambivalence arises when norms are contradictory, such as the norm for autonomy versus the norm of dependence for adult offspring (Luescher & Pillemer, 1998). And as I discuss, norms for autonomy contrast current interdependence in this tie, providing fodder for ambivalence. Moreover, frequent contact provides more opportunity for conflicts to arise (van Gaalen & Dykstra, 2010). Taken together, these trends suggest that intergenerational ambivalence between midlife parents and grown children also may be on the rise.

## Why Parent/Offspring Ties Have Changed

**Figure 1.**
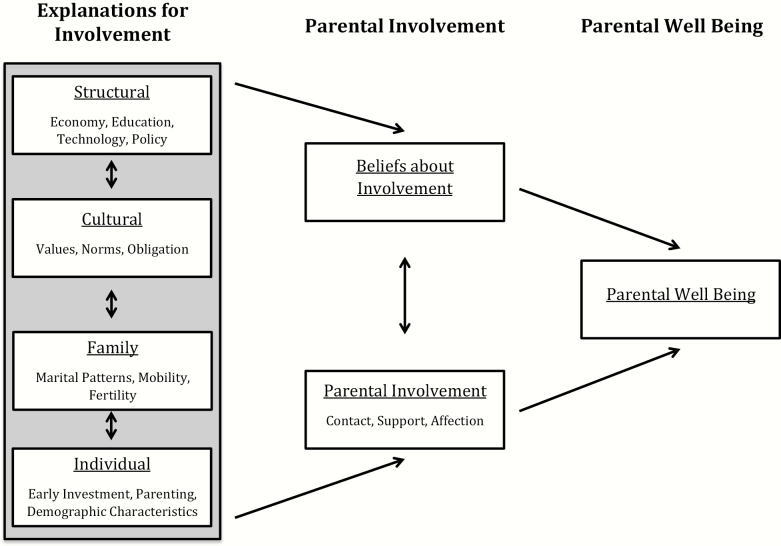
Multidimension intergenerational involvement model.

The *Multidimensional Intergenerational Support Model* (MISM) provides a framework to explain behaviors in parent/child ties. The model initially pertained to patterns of exchange between generations, but extends to a broader understanding of increased parental involvement. Drawing on life course theory and other socio-contextual theories, the basic premise of the MISM model is that structural factors (e.g., economy, technology, policy), culture (norms), family structure (e.g., married/remarried), and relationship and individual (e.g., affection, gender) factors coalesce to generate behaviors in intergenerational ties (Figure 1.) Likewise, changes in the parent/child tie and the reasons underlying those changes reflect such factors.

MISM is truly intended as a framework for stipulating the types of factors that contribute to parents’ and grown children’s relationship behaviors rather than a model of causal influences. Scholars interested in ecological contexts of human development have often designated hierarchies or embedding of different types of contexts (e.g., family subsumed in economy; Bronfenbrenner & Morris, 2006; Elder, 1998). Intuitively, young adults’ and midlife parents’ relationships do respond to economic factors, with the Great Recession partially instigating the increase in coresidence (Fry, 2015). Yet, economies arise in part from families and culture as well; in Western democracies, policies, and politicians are a reflection of underlying beliefs and values of the people who vote (as post-election dissection of Presidential voting in the United States suggests). As such, I propose that each of these levels—structural (e.g., economy, policy), cultural (beliefs, social position), family (e.g., married parents/single parent), and relationship or individual factors contribute to midlife parents’ involvement with grown children without implying a hierarchy of influence among the factors. As discussed later, a second aspect of Figure 1 pertains to understanding how parent/child involvement is associated with parental well-being.

### Societal Shifts Associated With Changes Between Parents and Young Adults

#### Economic factors

Economic changes in the past 40 years weigh heavily on the parent/child tie. Young adults’ dependence on parents reflects complexities of gaining an economic foothold in adulthood. The U.S. Census shows that financial independence is rare for young people today. Compared to their mid twentieth century counterparts, young people today are more likely to fall at the bottom of the economic ladder with low wage jobs. In 1975, fewer than 25% of young adults fell in the bottom of the economic ladder (i.e., less than $30,000 a year in 2015 dollars), but by 2016, 41% did (Vespa, 2017).

Further, roughly one in four young adults who live with their parents in the United States (i.e., 32% who live with parents; Fry, 2016) are not working or attending school (Vespa, 2017). These 8% of young adults might reside with parents while raising young children of their own. But notably, the rate of young women who were homemakers fell from 43% in 1975 to just 14% in 2016 (Vespa, 2017) and as I discuss later, fertility has also dropped in this age group (World Bank, 2017a). Moreover, a large proportion of young adults who live with parents have a disability of some sort (10%; Vespa, 2017). Thus, factors other than childrearing such as disability, addiction, or life problems seem more likely to account for the 2.2 million 25–34 year olds residing with parents not engaged in work or education.

Moreover, the shift toward coresidence with parents is not purely economic—one can imagine a society where young people turn to friends, siblings, or early romantic partnership to deal with a tough economy. Thus, other factors also contribute to these patterns.

#### Public policies

Public policies play a strong role in shaping relationships between adults and parents in European countries, but may play a lesser role in shaping these ties in the United States. In European countries, the government provides health coverage and long-term care, and government investments in older adults result in transfers of wealth to their middle generation progeny (Kohli, 1999). Similar processes occur with regard to midlife parents and young adults in Europe. Differences in programs to support young adults in Nordic countries versus Southern European countries are associated with the type of welfare state; that is, social democratic welfare regimes assist young adults in Nordic countries towards autonomy, whereas conservative continental or familistic welfare regimes encourage greater dependence on families in southern Europe (Billari, 2004). The coresidence patterns described previously conform to the type of regime. As such, patterns of parental involvement in Europe seem to be associated with government programs.

These patterns are less clear in the United States. Indeed, *lack* of government support for young adults may help explain many aspects of the intensified bonds. For example, as college tuition has increased and state and federal funding of education has decreased, parents have stepped in to provide financial help or co-sign loans for young adult students. When U.S. policies do address young adults, the policies seem to be popular. For example, in 2017, when the U.S. Congress debated repealing the Affordable Care Act (i.e., Obamacare), there was bipartisan support for allowing parents to retain grown children on their health insurance until age 26, even if these young adults were not students. This policy, instigated in 2011, seemed to be a reaction to the greater involvement of parents in supporting young adults rather than a catalyst of such involvement.

#### Education

Related to economic changes, a global rise in parental support of young adults may partially reflect the prolonged tertiary education that has occurred throughout the world (i.e., rates of college attendance have risen worldwide; OECD, 2016). In the United States, in 2016, 40% of adults aged 18–24 were pursuing higher education (National Center for Education Statistics, 2017), the highest rate observed historically. Similarly, in industrialized nations, young adults are more likely to attend college today than in the past (Fingerman, Cheng, et al., 2016).

The influence of education on parental involvement has been observed globally. In young adulthood, students receive more parental support than nonstudents (Bucx, van Wel, & Knijn, 2012; Johnson, 2013). A study of college students in Korea, Hong Kong, Germany, and the United States revealed that, across nations, parents provided advice, practical help, and emotional support to college students at least once a month (Fingerman et al., 2016). Young people who don’t pursue an education may end up in part time jobs with revolving hours or off hour shifts and may depend on parents for support (Furstenberg, 2010), but students typically receive more parental support (Henretta et al., 2012).

#### Technology and geographic stability

Recent technologies also have altered the nature of the parent/child bond, allowing more frequent conversations and exchanges of nontangible support (e.g., advice, sharing problems). Beginning in the 1990s, competitive rates for long distance telephone calls facilitated contact between young adults and parents who resided far apart. Since that time, cell phone, text messages, email, and social media have provided almost instantaneous contact at negligible cost, regardless of distance (Cotten, McCollough, & Adams, 2012).

Parents and grown children also may have more opportunities to visit in person. Residential mobility decreased in the United States from the mid-20th century into the 21st century. Data regarding how far young adults reside from their parents in the United States are not readily available. But in 1965, 21% of U.S. adults moved households; mobility declined steadily over the next 40 years and by 2016 had dropped to 11% (U.S. Census Bureau, 2011, 2016). As such, parents and grown children may be more likely to reside in closer geographic proximity. Deregulation of airlines in 1978 in the United States established the basis for airline competition and declining prices in airfare (with concomitant diminished quality of air travel experience), facilitating visits between parents and grown children who reside at longer distances.

### Cultural Beliefs Associated With Changes Between Parents and Young Adults

Culture also contributes to the nature of parent/child ties. Parents and grown children harbor values, norms or beliefs about how parents and grown children *should* behave. Shifts in cultural values have also contributed to increased involvement.

#### Historical changes in values for parental involvement

The cultural narrative regarding young adults and parents in the United States has shifted over the past few decades. During the 1960s and 1970s, popular media and scholars referred to the “generation gap” involving dissension between midlife parents and young adult children (Troll, 1972). This cultural notion of a gap reflected the younger generation’s separation from the older one during this historical period. For example, in 1960, only 20% of adults aged 18–34 lived with their parents (Fry, 2016). Into the 1970s, 80% of adults were married by the age of 30 (Vespa, 2017). As such, the generations were living apart. Cultural attention to a generation gap reflected the younger generation’s independence from the older generation. Notably, there was not much empirical evidence of generational *dissension*. And in the 21st century, this conception of separation of generations and intrafamily conflict seems antiquated.

Today’s cultural narrative is consistent with increased intimacy and dependence of the younger generation, while also disparaging this increased parental involvement. Recent media trends and scholarly work in the early 21st century focus on “helicopter parents” who are *too* involved with their grown children (Fingerman, Cheng, Wesselmann, et al., 2012; Luden, 2012). Although the concept of the helicopter parent implies intrusiveness, it is also a narrative that reflects increased contact, intimacy, and parental support documented here. The pejorative aspect of the moniker stems from retention of norms endorsing autonomy; the relationships are deemed *too* close and intimate. Although intrusive parents undoubtedly exist, there is little evidence that intrusive helicopter ties are pervasive (outside small convenience studies of college students). Rather, young adults seem to benefit from parental support in many circumstances (Fingerman, Cheng, Wesselmann, 2012), but to perhaps question their own competency under some circumstances of parental support (Johnson, 2013). Nevertheless, a cultural lag is evident in beliefs about autonomy in young adulthood versus the increased parental involvement. Many midlife parents believe young adults should be more autonomous than they are (Fingerman, Cheng, Wesselmann, et al., 2012).

#### Historical changes in sense of obligation

Shifts in beliefs are notable with regard to a diminished sense of obligation to attend to parent/child ties as well. Obligation has been measured most often with regard to midlife adults’ beliefs concerning help to aging parents (i.e., filial obligation). For example, Gans and Silverstein (2006) examined four waves of data regarding adults’ ties to parents from 1985 to 2000; they documented a trend of declining endorsement of obligation over that period. Similarly, many Asian countries (e.g., China, Korea, Singapore) traditionally followed Confucian ideals involving a high degree of respect and filial piety. But over the past three decades, these values have eroded in these countries (Kim, Cheng, Zarit, & Fingerman, 2015). As such, norms obligating parent/child involvement seem to be waning.

Instead, the strengthened bonds and increased parental involvement may reflect a *loosening* of mores that govern relationships in general. Scholars have suggested that increased individual freedom and fewer links between work, social activity, and family life characterize modern societies over the past decades. These changes also are associated with evolving family forms (e.g., divorce and stepties) as well as decreased fertility (Axinn & Yabiku, 2001; Lesthaegh, 2010). Likewise, this loosening of rules has rendered the parent/child relationship more chosen and voluntary in nature. This is not to say the tie has become reciprocal; parents typically give more to offspring than they receive (Fingerman et al., 2011). Yet, the increased involvement and solidarity may stem from freedom parents and grown children experience to retain strong bonds (rather than following norms of autonomy).

#### National and ethnic differences in beliefs about parent/child ties

The role of beliefs and values in shaping ties between young adults and parents is evident in cross national differences. High parental involvement occurs most often in cultures where people highly value such involvement. Analysis of European countries has found that in countries where adults and parents coreside more often, adults place a higher value on parental involvement with grown children (Hank, 2007; Newman, 2011). For example, families in Southern Europe (Spain, Italy, Greece) coreside most often and also prefer shared daily life. Based on this premise, we would expect to see a surge in norms in the United States endorsing intergenerational bonds and young adults’ dependence on parents, but this is not necessarily the case.

In addition to the cultural lag mentioned previously, within the U.S. ethnic differences in parental beliefs about involvement with young adults are evident. For example, Fingerman, VanderDrift, and colleagues (2011) examined three generations among Black and non-Hispanic White families. Findings revealed that overall, non-Hispanic White midlife adults provided more support of all types to their grown children than to their parents. Black midlife adults also provided more support overall to their grown children than to their parents, but they provided more emotional support, companionship, and practical help to their parents. Importantly, midlife adults’ support to different generations was consistent with ethnic/racial differences in value and beliefs—Black and non-Hispanic adults’ support behaviors were associated with their perceived obligation to help grown children and rated rewards of helping grown children and parents (above and beyond factors such as resources, SES, offspring likelihood of being a student, and familial needs) (Fingerman, VanderDrift, et al., 2011). These findings were consistent with a study conducted in the late 20th century using a national sample of young adults; that study found that racial and immigration status differences in parents’ support of young adults reflected factors in addition to young adult resources, family SES, or other structural factors (Hardie & Selzter, 2016), presumably cultural differences. As such, the overall culture surrounding young adults and family may play a role in increased parental involvement.

### Family Factors Associated With Changes Between Parents and Young Adults

Changes in family structure are likely to affect the nature of parent/child relationships, including (a) proportion of mothers married to a grown child’s father, (b) likelihood of a midlife parent having stepchildren, and (c) the grown child’s fertility. Collectively, these family changes contribute to the nature of bonds between young adults and parents, and raise questions about the future of this tie.

#### Declines in married parents and rise of stepfamilies

Changes in parents’ marital status contribute to relationships with grown children in complex ways. Some changes facilitate the strengthened bonds observed, but other changes diminish the likelihood of a strong bond. As such, while the overall trend shows greater parental involvement, specific groups of midlife parents may have only tenuous or conflicted ties with their grown children.

The previous few decades saw a shift from families where two parents were likely to be married to one another toward single parents and complex family forms. From 1970 to 2010, the marriage rate for women in the United States declined steadily, particularly for Black women (in 2010 only 26% of Black women were married; Cruz, 2013). Mothers who raise children alone typically have stronger ties when those children grow into young adults. By contrast, never-married fathers may have little contact and are more likely to be estranged from those children (Hartnett et al., 2017).

Further, midlife adults are more likely to have ties to grown children through remarriage (i.e., stepchildren) than in the past. Divorce rates rose and plateaued in the mid to late twentieth century. Divorce is associated with greater tensions between young adults and parents, particularly for fathers (Yu, Pettit, Lansford, Dodge, & Bates, 2010).

Remarriage rates also continued to rise over the past few decades; 40% of all marriages involve at least one partner who was previously married (Livingston, 2014). A recent survey found 18% of adults in the United States aged 50–64 and 22% of adults over age 65 had a stepchild (Pew Research Center, 2011). Stepparents are less involved with grown stepchildren (Aquilino, 2006) and feel less obligated to help stepchildren than biological/adoptive parents do (Ganong & Coleman, 2017; Pew Research Center, 2011). Thus, many midlife adults have ties to grown children that do not involve the intensity of biological relationships. Yet, it is not clear whether these same midlife adults have biological children to whom they remain close.

#### Young adults’ marriage and fertility

Young adults’ marital and procreation patterns may contribute to more intense bonds with midlife parents. In well-off families, young adults are delaying marriage (Cherlin, 2010). Given that marriage typically draws young adults away from parents (Sarkisian & Gerstel, 2008), this delay may contribute to more intense ties with parents. Upper SES young adults are more likely to marry, but do so at later ages (Vespa, 2017) and thus, also retain stronger ties to parents.

Changes in childbearing also may facilitate prolonged ties to parents. The transition to adulthood co-occurs with the period of highest fecundity, but several factors contribute to diminished fertility since 1960s (World Bank, 2017a). Rising levels of women’s education and effective contraception are associated with lower birth rates (Lesthaegh, 2010). Americans no longer believe parenthood is a key marker of adulthood (Vespa, 2017). Further, declines in fertility occur during economic downswings, such as the Great Recession (Mather, 2012).

Declines in fertility lengthen the period of time in which young adult retain child-free ties to parents, and also shape the midlife adults’ transition to grandparenthood. Yet, the likelihood and experience of being a grandparent also differs by socioeconomic position. In lower SES families, young adult women are more likely to become mothers without a long term partner (Cherlin, 2010); their midlife mothers (the grandmothers) may help with childcare, housing, and other support. Further, lower SES midlife parents are more likely to be involved in living with or raising grandchildren (Ellis & Simons, 2014; U.S. Census Bureau, 2014). Thus, a majority of midlife adults remain in limbo with regard to whether and when they will become grandparents and their involvement with their own children reflects a prolongation of prior parental involvement, but a subset of typically under-privileged midlife parents may be highly involved in care for grandchildren.

### Relationship and Individual Characteristics Associated With Parent/Child Ties

Finally, ties between midlife adults and their grown children occur between two people, and the characteristics of these people and their shared history account for the nature of those relationships.

#### History of the relationship

Close relationships in young adulthood may arise from strong relationships in childhood and adolescence. Attachment theory suggests children form bonds to parents in infancy that endure into their relationship patterns in adulthood, and theorists also argue that parents retain bonds to children formed earlier in life (Antonucci & Akiyama, 1994; Kahn & Antonucci, 1980). Of course, these assumptions raise questions about what types of relationships are likely to be stronger in childhood and adolescence.

Similar structural, cultural, and family contexts contribute to childhood patterns and to continuity into adulthood. For example, upper socioeconomic status parents are more likely to engage in intensive parenting when their children are young such as playing games with them and ferrying them to soccer practice (Bianchi & Milkie, 2010; Sayer, Bianchi, & Robinson, 2004). Likewise, parental marital status plays a role in these patterns, with divorced or single fathers less involved with young children than coresident married fathers (Kalmijn, 2013a; Sweeney, 2010). Lower socioeconomic mothers may be involved with their children because they are more likely to be never married or divorced. A complete review of the factors that shape ties between young children and parents is beyond the scope of this article, but suffice it to say that the factors that account for ties between young adults and parents also shape ties earlier in the lifespan, and that observed relationships between young adults and parents in part arise from these earlier relationships.

### Individual Characteristics and Within Family Differences

In addition, midlife parents bring individual characteristics to their relationships with grown children, including their gender, socioeconomic position, and marital status. Socioeconomic position has already been covered with regard to provision of support, and marital status was reviewed with regard to family structure.

But parental gender also plays a key role, favoring maternal involvement with grown children. The pattern of current maternal involvement is not new; research from the mid twentieth century documented that mothers were consistently more involved than fathers were with grown children of all ages (Rossi & Rossi, 1990; Umberson, 1992).

Parental gender is situated in a variety of other contextual variables, including SES (single mothers likely to be poorer, with fewer financial resources for children) and marital status (e.g., unmarried mothers are closer to their grown children, unmarried/remarried fathers have lessened involvement or may be estranged from grown children). Yet, studies find that mothers have more frequent contact with grown children, provide more support, and report greater closeness and conflict at midlife even after controlling for social structure and marital status (e.g., Arnett & Schwab, 2012a; Fingerman et al., 2009; Fingerman et al., 2016).

Notably, relationships between young adults and parents also vary *within* families. That is, parents do not have equally intense relationships with each of their children (Suitor et al., in press). Parents respond to their children’s characteristics and their sense of compatibility with each child. Parents provide support in reaction to crises (e.g., divorce, illness) or ongoing everyday needs associated with a child’s statuses (e.g., child is a parent; student) or age (Hartnett et al., 2017). Parents also are more likely to give support to young adult and midlife children whom they view as successful, with whom they have closer relationships, or with whom they share values (Kalmijn, 2013b; Suitor, Pillemer, & Sechrist, 2006; Suitor et al., 2016).

Declining fertility described previously may diminish within-family variability in the future (World Bank, 2017a). Today’s midlife adults grew up in larger sibships than today’s young adults, and parents invest more in each child in smaller sibships (Fingerman et al., 2009). As such, the intensity of ties between midlife parents and their grown children is generally higher than in the past, and likely to remain high, with diminishment of within family variability.

## Implications of Changes in Young Adulthood for Midlife Parents’ Well-Being

All of these issues raise the question—do changes in parents’ ties to young adults matter for the parents? Theory and research regarding the effects of parental involvement have focused on the grown child (e.g., Johnson, 2013) rather than on the parent.

Emerging evidence suggests involvement with young adult offspring has implications for midlife parents’ current well-being, however. The research literature on this topic is nascent, beginning in the past 10 years (perhaps reflecting the increase in parental involvement during that period). Further, most studies examine effects of parental involvement without contextual factors such as SES or marital status. As such, the MIS model (Figure 1) is comprised of two models, one model predicting parental involvement from a variety of factors, and the other model predicting parental well-being from parental involvement. Several of the connections between levels of the model are theoretical and warrant additional research attention. In describing associations between parental involvement and well-being, I highlight which factors might warrant particular research attention in the future.

### Generativity and benefits of parental involvement

Midlife parents may benefit from involvement with their grown children. Erikson’s (1963) theory of lifespan development indicated the task of midlife is generativity—that is, midlife adults derive rewards from giving to the next generation. In the context of the parent/child tie, one study found that parents who gave more instrumental support to their grown children reported better well-being (fewer depressive symptoms) over time (Byers, Levy, Allore, Bruce, & Kasl, 2008). Similarly, another study found that parents shared laughter and enjoyable exchanges with grown children in their daily interactions. Over the course of the study week, 90% of the parents (*N* = 247) reported having an enjoyable encounter with a grown child, and 89% reported laughing with a grown child (Fingerman, Kim, Birditt, & Zarit, 2016).

Yet, not all parents experience such generativity and enjoyment of grown children. The family factors described previously may play a role in whether parents benefit from, or are harmed by, involvement with grown children. Parents who are estranged from offspring (i.e., fathers) may suffer diminished well-being due to the loss of this normative role. Similarly, stepparents may incur fewer rewards due to lessened involvement with grown children. Future research should focus specifically on opportunities for generativity in different populations, particularly among midlife men.

Further, as mentioned, midlife adults are less likely to be grandparents due to young adults’ delayed fertility (or decisions to not have children). Midlife adults who are grandparents are often highly involved with their grandchildren (as well as their grown children), providing childcare on a frequent basis (Hank & Buber, 2009). Grandparents typically find the grandparenting role rewarding (Fingerman, 1998). Future research should ask whether midlife adults who have grown children, but not grandchildren experience frustration or longing.

### Emotional involvement and grown children’s problems

Parental well-being also may align with events in their grown children’s lives. Coregulation of emotions has been found in marital couples and in ties between parents and younger children who live in their home (Butler & Randall, 2013). Likewise, the increased frequency of contact with grown children may generate an immediate emotional response to problems grown children experience. Indeed, factors that have facilitated contact between generations, such as technologies, decreased mobility, and coresidence allow parents to experience immediate reactions to events in grown children’s lives. For example, in the 1980s, a grown child who failed a college exam might call at the end of the week to relate that story to a parent, along with the resolution of the problem (the professor offered extra credit because students did not perform well on that test). The parent learned of the events without reacting emotionally. By contrast, in the 21st century, young adults text or call their parents in the throes of crisis, and parents experience the vicissitudes of young adulthood in the moment.

In particular, midlife parents incur detriments from grown children suffering life crises such as divorce, health problems, job loss, addiction, or being the victim of a crime. Researchers have found that even one grown child experiencing one problem has a negative effect on a midlife parent, regardless of how successful other children in the family might be (Fingerman, Cheng, Birditt, & Zarit, 2012). Similarly, in late life, mothers suffer when grown children experience such crises, irrespective of their favoritism or feelings about the grown child (Pillemer, Suitor, Riffin, & Gilligan, 2017). These effects on parental well-being may reflect a variety of responses including a sense that one has failed in the parenting role, worry about the child, empathy with the grown child, or stress of trying to ameliorate the situation (Fingerman, Cheng, Birditt, et al., 2012; Hay, Fingerman, & Lefkowitz, 2008). Again, structural factors such as SES are associated with the likelihood parents will have a grown child who experiences such problems. That is, lower SES is associated with increased risks of a grown child experiencing financial and other life problems.

The familial changes noted previously also may play a role regarding which parents are affected by grown children. Stepparents may incur fewer rewards from stepchildren and less harm when their stepchildren suffer problems compared to biological (or adopted early in life) children. Yet, the marriage may suffer if the stepparent objects to the biological parents’ involvement with a grown children who has incurred a life crisis. Future research should address these issues.

In sum, many midlife parents incur benefits from their stronger ties to grown children. But when grown children experience life crises—job loss or serious health problems—these problems may undermine their parents’ well-being, particularly when parents are highly involved with those grown children.

### Beliefs About Involvement With Grown Children

Parents’ beliefs about their involvement with grown children may also be pivotal in the implications of that involvement for their well-being. Cognitive behavioral theories suggest that individuals’ perspectives on these relationships determine the implications of involvement with family members. Indeed, research regarding intergenerational caregiving has established that beliefs about the caregiving role and subjective burden contribute to the implications of caregiving more than the objective demands of caregiving (Aneshensel, Pearlin, Mullan, Zarit, & Whitlatch, 1995; Zarit, Reever, & Bach-Peterson, 1980).

Similar processes may be evident regarding midlife parents’ involvement with their grown children. It is not so much the involvement, per se, as the parents’ perceptions of that involvement that affects the parents’ well-being. For example, in one study, when midlife parents provided support to grown children several times a week, parents’ ratings of the child’s neediness were associated with parental well-being. Parents who viewed their grown children as more needy than other young adults reported poorer well-being, but the frequency of support the parents provided was not associated with the parents’ well-being (though more frequent support was beneficial from the grown child’s perspective; Fingerman, Cheng, Wesselmann, et al., 2012).

Shifts in beliefs and the associations with well-being may reflect both the overall cultural norms for parental involvement and the economy. For example, a study in the United States before the Great Recession (when intergenerational coresidence was less common) found that adults of all ages endorsed coresidence between generations solely when the younger generation incurred economic problems or was single and childless (Seltzer, Lao, & Bianchi, 2012). A more recent study of the “empty nest” found that midlife parents who had children residing in their home in 2008 had poorer quality marital ties. But in 2013 (when intergenerational coresidence became more common), parents residing with offspring reported poorer marital quality only when their children suffered life problems (Davis, Kim, & Fingerman, in press). Thus, norms for parental involvement with grown children and the economic context may shape the implications of that involvement for parents’ marital ties and well-being. Parents are harmed when they believe their grown children should be more autonomous (Fingerman, Cheng, Wesselmann, et al., 2012; Pillemer et al., 2017).

## Future Consequences of Today’s Young Adulthood for Parents Entering Late Life

Given the implications of young adult children for midlife parents’ well-being, it is worth considering how relationships with grown children may shape parents’ later years. We might consider two possible pathways with regard to parental aging. First, parents may continue in the role of parenting by giving support to the next generation even as the offspring transition to midlife. Second, most parents will require assistance at some point in the aging process. Again, the economic structures, norms, and family structures evident today may shape these processes, but the research is not well-developed regarding variability in these patterns.

### Continued Involvement in the Parenting Role

Given current patterns of heavy involvement, parents may persist in the parenting role into late life. Primates demonstrate a general parenting predisposition long past the years of the progeny’s immaturity (at least among mothers). Jane Goodall, the famous primatologist reported her observations of Flo, an elderly female chimp. One day, Flo viciously attacked a young male chimp who had engaged in a fight with her son, Figan. Despite the aged Flo’s weaker status, she jumped in to protect her grown offspring (Montgomery, 2009).

Human “primates” behave in a similar manner, continuing in the parental role and providing for their children in need, even in late life (Suitor et al., 2006; Suitor, Sechrist, & Pillemer, 2007). These patterns are evident across cultural groups. Research regarding Western countries throughout Europe found that parents gave more support to grown children than the reverse (Grundy & Henretta, 2006; Kohli, Albertini, & Kunemond, 2010). In the United States, Becker, Beyene, Newsom, and Mayen (2003) conducted a qualitative study of family ties among older adults in four different ethnic groups (Latino, African American, Vietnamese, and Filipino). Although the scholars noted ethnic differences in how groups viewed coresidence and family ties, older relatives in all four groups attempted to give financial or practical help such as child care to the younger generation.

Moreover, although Asian cultures have traditionally endorsed Confucian values for grown children to provide support *to* parents (Kim et al., 2015), research in China finds that rural older adults still provide practical support to grown children or childcare for their grandchildren (i.e., if the grandchildren’s parents move to urban areas; Chen & Silverstein, 2000). Thus, despite cultural and economic differences, overall parents may remain heavily invested in the parenting role into late life and are likely to do so in the future.

### Parental Needs for Care

Nevertheless, parents also typically incur needs for support by the end of life when physical health or cognitive abilities decline. Midlife children have been a mainstay of this support. As such, we ask how today’s young adults will care for their parents in old age.

The strengthening of intergenerational bonds may serve many older parents well at the end of life. Research examining current cohorts of older adults suggests that aging parents are more likely to receive care from a child who shares their values and with whom they have had a close relationship (Pillemer & Suitor, 2013). Extending this pattern into future cohorts, the prolonged transition to adulthood provides opportunities for parents and young adult children to develop strong bonds. By midlife, these ties may facilitate a seamless transition to caregiving tasks because the two parties already engage in daily exchanges of emotional and practical help (Fingerman et al., 2016; Fingerman, Huo, Kim, & Birditt, in press).

By contrast, prolonged dependency on parents may stymie the offspring’s psychological growth and could impede the ability of midlife adults to care for their parents in late life. Researchers have shown that investment in adult roles (e.g., work, family) is associated with personality changes consistent with providing care to others (e.g., increased agreeableness, conscientiousness, emotional stability; Bleidorn et al., 2013; Lodi-Smith & Roberts, 2007). As such, exclusion from these roles may diminish psychological growth associated with helping parents in late life.

Yet, evidence suggests offspring will step in when the time come based on current patterns. A survey of over 1,000 caregivers in the United States revealed that nearly a quarter of them were aged 18–34 (AARP, 2015). Granted, these younger caregivers typically were involved as secondary caregivers and put in fewer hours than older adults who were caring for a spouse. Nevertheless, these data suggest millennials are already stepping in to care for family.

Of course, patterns may vary within and between families. Some of the best predictors of relationship qualities in parent/child relationships over time are prior relationship qualities (Belsky, Jaffee, Shieh, & Silva, 2001; Suitor, Gilligan, & Pillemer, 2013). In essence, it is likely that parent/child ties that are well-functioning in young adulthood may persist in this manner, providing excellent care to aging parents. By contrast, relationships that are already fraught with difficulties may disband or generate inadequate parent care in late life.

#### Factors associated with future support

The factors that underlie current parental involvement may also shape the likelihood and type of future support that parents receive in old age. Yet structural factors may evolve over time. Thus, for any individual midlife parent today, future circumstances may be different.

Regarding socioeconomic status, prolonged parental support of young adults may have implications for parents’ financial well-being, even among parents who are not badly off today. Money is finite. As such, when midlife parents provide financial support to grown children, that support may come at the expense of the parents’ current and future financial well-being (e.g., own retirement savings). These patterns may be exacerbated for Americans in the bottom half of the economic ladder, who are unlikely to save for retirement at all (Rhee, 2013). Yet, it is not clear how current financial demands on midlife parents bode for the future. For example, coresidence is more common among lower SES parents and adult children. And this coresidence may be setting up patterns now that *facilitate* support of parents in late life. A recent survey found that nearly half of grown children who reside with parents paid rent and nearly 90% contributed to household expenses (Pew Research Center, 2012). When parents age, these children may step in with financial support providing lower SES parents with a safety net.

The role of technology in future ties with aging parents also is unclear. The technological advances of the early 21 century facilitated communication between adults and parents, as cell phones saturated markets nearly worldwide by 2015 (World Bank, 2017b). Yet, as the 21st century unfolds, social media are a dominant force in communication patterns. More importantly, social media platform usage differs by cohort. For example, 62% of adults who are online use Facebook, but young adults are more likely to use Instagram (59% of adults aged 18–29 in 2015 used Instagram, compared to only 8% of older adults; Pew Research Center, 2016). If these patterns persist, by late life, current midlife parents who use a certain form of social media may be shut out of communication if their grown children use a different social media platform. Perhaps this alienation will be avoided if both parties use a single social media platform—even a new one that emerges in the future.

Family structure may also have implications for parents as they grow older and require care. In the 20th century, scholars debunked the idea of the “sandwich generation” as a falsehood; most midlife caregivers had children who were grown and were not raising young children while caring for aging parents (Fingerman et al., 2010; Grundy & Henretta, 2006). If anything, today’s midlife adult is likely to be in a “club sandwich” where they confront demands from layers of generations—caregiving for an aging parent and responding to crises and everyday needs among their young adult offspring in sequence and simultaneously (Fingerman, Pitzer, et al., 2011; Grundy & Henretta, 2006).

For young adults today who have children at later ages, however, a true sandwich may occur, with both generation pressing on the midlife generation squished like jelly in between. Aside from the increased stress on the midlife caregiver, quality of care may suffer. Older parents may worry about burdening their grown children and may not ask for help they need. Even older parents who seek assistance may suffer due to constraints on the midlife child who is consumed with raising her own children. In some families, adolescent grandchildren may supplement care provided by a midlife adult (AARP, 2015; Hamil, 2012), and future research should examine this type of supplementary care. In sum, in the future, parents may find that their midlife children are experiencing strains balancing children in the home and aging parents, but some families may come together in caregiving with a third generation joining in.

Finally, with declining fertility rates, parents may selectively turn to midlife children who lack children of their own for care. Yet, the evidence for this assertion is mixed. In one study, aging mothers identified the grown child they anticipated would provide care and then examined who actually did provide care 7 years later. That study found no such association regarding whether the midlife child had children of his/her own (Pillemer & Suitor, 2013), perhaps because adults who have children of their own assume nurturing roles toward their parents as well as their children. Other research has found that gay and lesbian married couples do a better job of supporting one another when providing care to aging parents than do heterosexual marital couples (Reczek & Umberson, 2016). The authors attributed some of this spousal support of caregiving to gendered roles in marriage (i.e., men expect women to do caregiving but this not the case in gay and lesbian couples). Still, heterosexual couples are also more likely to have children who generate additional burdens competing with parental caregiving. Future research will need to examine how the current generation of parents elicits care from their offspring who may or may not have children of their own.

## Directions for Future Research and Conclusions

Parents are considerably more involved with their grown children aged 18–34 than was the case 40 years ago. Parents engage in more frequent contact, give more support, are more likely to live with a grown child, and experience greater affection. Societal changes in the form of economic challenges to attaining adulthood, new technologies facilitating communication, and public policies that place greater reliance on family contribute to these stronger bonds.

Distinct subgroups of parents warrant additional research attention. For example, although LGBT youth have received research attention—particularly with regard to coming out to parents (Pew Research Center, 2013), studies examining LGBT midlife parents and their young adult offspring are all but absent from the literature. It is likely that these relationships are as involved as relationships involving married or single heterosexual parents, but the history of discrimination and inability to marry earlier in life may offer unique features to these ties.

Moreover, cultural values and beliefs are in flux. Many parents in the United States continue to endorse beliefs about grown children’s autonomy (Fingerman, Cheng, Wesselman, et al., 2012; Vespa, 2017). It is not clear when (and whether) those values will shift and future studies should focus on this issue. Research should also seek to understand parental beliefs about goals during the adolescent years and the types of tasks parents expect their adolescent children to perform to prepare for young adulthood.

Of equal concern is the dearth of *recent* data regarding ethnic and racial differences in parents’ ties to young adults. Many publications regarding ethnic and racial differences among parents and young adult children still analyze data from the 1990s (e.g., Hardie & Seltzer, 2016), and it is not clear whether the findings are relevant in 2017.

Finally, there has been little attention to potential distinctions between rural and urban settings with regard to the transition to adulthood, and young adults’ ties to their midlife parents. Studies have examined these differences in China where urban residence requires permits that aging parents often lack (Chen & Silverstein, 2000). Given the outflux of young people from rural areas in the United States, this topic warrants consideration in the United States as well.

The penultimate issue is how ties between midlife parents and young adult children will evolve into tomorrow’s support for aging parents. Rather than judge or criticize current patterns (e.g., young adults are immature), future research might seek to identify how the strengths of current patterns could lead to support of aging parents. Indeed, decades of research addressing marriage has generated algorithms to predict divorce and to provide interventions for maladaptive marital relationship patterns. Similar initiatives may be warranted with regard to intergenerational ties which seem to be replacing romantic partnerships as the primary relationships for many adults today.

Anecdotally, young adults seem to be involved in ways that facilitate their midlife parents’ well-being in many situations today. When Hurricane Harvey hit the Gulf Coast during the first week of classes at UT Austin in late August, 2017, the Provost sent a memo to faculty asking them to be flexible for the many students from Houston affected by the hurricane. In that memo, the Provost correctly pointed out that many of these young people would spend the semester going back and forth to Houston to help their parents move in and out of shelters and to restart their lives. This disaster brought to light the ways in which millennials reciprocate their parents’ involvement and investment via a strong sense of family cohesion in return.

In conclusion, involvement with young adult children has ramifications for midlife parents in positive and negative ways. Parents benefit from a close tie with frequent contact, and many parents find it rewarding to be involved in their grown children’s lives. Parents may also suffer if they vicariously experience their children’s life crises. Nevertheless, parental involvement may help mitigate children’s crises and improve the parents’ well-being as a result. And the offspring may step up and be there in moments of crisis as well. In sum, most parents view their grown children as valuable relationship partners from whom they benefit in the present, and may benefit in the future.

## Funding

This study was supported by grants from the National Institute on Aging (NIA), National Institutes of Health (R01AG027769) the *Family Exchanges Study II* to K. L. Fingerman, Principal Investigator. This research also was supported by grant (5 R24 HD042849) awarded to the Population Research Center (PRC) at The University of Texas at Austin by the Eunice Kennedy Shriver National Institute of Child Health and Human Development (NICHD), National Institutes of Health.

## Conflict of Interest

None reported.
